# Longitudinal analysis of the antibody repertoire of a Zika virus-infected patient revealed dynamic changes in antibody response

**DOI:** 10.1080/22221751.2019.1701953

**Published:** 2020-01-06

**Authors:** Xuefeng Niu, Qihong Yan, Zhipeng Yao, Fan Zhang, Linbing Qu, Chunlin Wang, Chengrui Wang, Hui Lei, Chaoming Chen, Renshan Liang, Jia Luo, Qian Wang, Lingzhai Zhao, Yudi Zhang, Kun Luo, Longyu Wang, Hongkai Wu, Tingting Liu, Pingchao Li, Zhiqiang Zheng, Yee Joo Tan, Liqiang Feng, Zhenhai Zhang, Jian Han, Fuchun Zhang, Ling Chen

**Affiliations:** a State Key Laboratory of Respiratory Disease, The First Affiliated Hospital of Guangzhou Medical University, Guangzhou, People’s Republic of China; b Guangdong Laboratory of Computational Biomedicine, Guangzhou Institutes of Biomedicine and Health, Chinese Academy of Sciences, Guangzhou, People’s Republic of China; c University of Chinese Academy of Science, Beijing, People’s Republic of China; d Institute of Physical Science and Information Technology, Anhui University, Hefei, People’s Republic of China; e HudsonAlpha Institute of Biotechnology, Huntsville, AL, USA; f Department of Cardiology, State Key Laboratory of Organ Failure Research, Nanfang Hospital, Southern Medical University, Guangzhou, People’s Republic of China; g Guangzhou Eighth People’s Hospital, Guangzhou Medical University, Guangzhou, People’s Republic of China; h Department of Microbiology and Immunology, Yong Loo Lin School of Medicine, National University Health System (NUHS), National University of Singapore, Singapore, Singapore

**Keywords:** Zika virus, antibody, repertoire, monoclonal antibody, next-generation sequencing

## Abstract

The Zika virus (ZIKV) is a mosquito-borne flavivirus that causes neonatal abnormalities and other disorders. Antibodies to the ZIKV envelope (E) protein can block infection. In this study, next-generation sequencing (NGS) of immunoglobulin heavy chain (IgH) mRNA transcripts was combined with single-cell PCR cloning of E-binding monoclonal antibodies for analysing antibody response in a patient from the early stages of infection to more than one year after the clearance of the virus. The patient's IgH repertoire 14 and 64 days after symptom onset showed dramatic dominant clonal expansion but low clonal diversity. IgH repertoire 6 months after disease-free status had few dominant clones but increased diversity. E-binding antibodies appeared abundantly in the repertoire during the early stages of infection but quickly declined after clearance of the virus. Certain VH genes such as VH5-10-1 and VH4-39 appeared to be preferentially enlisted for a rapid antibody response to ZIKV infection. Most of these antibodies require relatively few somatic hypermutations to acquire the ability to bind to the E protein, pointing to a possible mechanism for rapid defence against ZIKV infection. This study provides a unique and holistic view of the dynamic changes and characteristics of the antibody response to ZIKV infection.

## Introduction

The Zika virus (ZIKV) is a member of the *Flaviviridae* family mainly transmitted by *Aedes* mosquitoes [1]; however, it is also reported that the virus can be transmitted through both sexual contact and blood transfusions [2,3]. Clinical evidence shows that ZIKV can cross the placental barrier and cause microcephaly in developing foetuses and neurological complications in adults such as Guillain-Barre syndrome [4,5]. The ZIKV envelope (E) protein mediates viral attachment to the host cell and fusion with cell membranes [6]. The flavivirus E ectodomain has three distinct domains: EDI-III [7]. Several ZIKV-specific antibodies have been isolated from infected individuals [8–12]; among these, E-binding antibodies, especially those targeting EDIII, manifest the most potent neutralizing activities *in vitro* and *in vivo* [8–11]*.* The presence of EDIII-targeted antibodies correlates with serum-neutralizing activity against ZIKV [8].

The human adaptive immune system consists of B and T cells, both of which play important roles in the defense against infections. A successful antibody response relies on the generation of a diverse repertoire of B cell receptors (BCRs). BCRs are membrane-bound antigen-binding immunoglobulins (Ig) that, like any antibody, are composed of two immunoglobulin heavy chains and two light chains. BCR diversity is achieved by rearrangement of the variable (V), diversity (D), and joining (J) gene segments in the immunoglobulin heavy chain locus (IgH) and the V and J gene segments in the light chain locus (Igλ or IgK) [13]. B cells are activated upon encountering an antigen that binds to their BCRs. Once a B cell encounters an antigen, it is recruited to a local lymph follicle and undergoes a process called somatic hypermutation (SHM), which increases the antigen-specific affinity in the germinal centres [14]. SHM occurs in a specific region, the complementary determining region (CDR), which is critical to antigen binding. The CDR is divided into three sub-regions: CDR1, CDR2, and CDR3. CDR3 typically plays a key role in determining antibody specificity and affinity [15]. Therefore, the CDR3 sequence is often used for lineage structure analysis of the antibody repertoire [16,17]. Activated B cells proliferate and differentiate to generate a population of antibody-secreting plasma B cells and memory B cells. Antigen-specific memory B cells in humans peak 14–21 days after infection or vaccination and can account for approximately 1% of all B cells in the peripheral blood [18]. Memory B cells can differentiate into both long-lived memory B cells, which mediate a rapid recall response, and long-lived plasma cells, which maintain antibody production [13].

Recently, next-generation sequencing (NGS)-based antibody repertoire analysis has been used to provide a systemic view of humoral responses to antigen stimuli [13] such as viral infections [19] and vaccination [17,20,21]. Understanding the mechanism and dynamics underlying antibody generation can facilitate vaccine design and improve the prognosis of infectious diseases [13,22]. In this report, we isolated E-binding mAbs from a ZIKV-infected patient and performed NGS analysis of the IgH mRNA repertoires to gain insight into the dynamics of the antibody response after infection.

## Materials and methods

### Human subject and peripheral blood cell isolation

The patient was a 28-year-old male who returned to Guangzhou from Venezuela in February 2016. He was hospitalized in Guangzhou 8th People’s Hospital in Guangzhou, China [10,23,24]. ZIKV RNA was detected in his serum, saliva, and urine samples by RT–PCR. The patient manifested relatively mild symptoms including fever, rash, sore throat, and fatigue. ZIKV was no longer detectable in the patient’s urine 14 days after symptom onset. He recovered completely and was discharged from the hospital after approximately three weeks. The patient tested serologically negative for DENV1 NS1, indicating that he had no previous exposure to DENV1 [10]. Whole blood samples measuring 8, 6, 8, and 8 mL (containing 4.8–6.4 million mononuclear cells) were collected from the patient at 14, 64, 181, and 412 days, respectively, into EDTA anticoagulant-containing tubes. Peripheral blood mononuclear cells (PBMCs) were isolated by density gradient separation on a Ficoll-Hypaque gradient (GE Healthcare, Chicago, IL, USA). All plasma samples were heat-inactivated at 56°C for 30 min prior to aliquoting and storage at – 80°C. This study was conducted in accordance with the rules and regulations stipulated by the Chinese government for the protection of human subjects. Furthermore, the patient provided written informed consent approving the use of his blood samples in the present study.

### Single B cell sorting, RT–PCR, sequencing, cloning, and expression of mAbs

Freshly isolated PBMCs were stained with a cocktail of the following antibodies: anti-human CD20-FITC (Invitrogen/Thermo Fisher Scientific, Carlsbad, CA, USA); IgG-APC-H7/CD3-Pacific Blue/CD27-PerCP-Cy5.5 (BD Biosciences, San Jose, CA, USA); and anti-HIS-PE (Miltenyi Biotec, Bergisch Gladbach, Germany). ZIKV E protein (Cat. No. 40543-V08B4; Sino Biological, Inc., Beijing, China) was used as a probe to isolate ZIKV E-specific memory B cells. CD3-CD20 + CD27 + IgG + HIS+ memory B cells were sorted using a multi-laser BD FACSAria™ II cell sorter (BD Biosciences). Individual B cells were sorted into 96-well PCR plates containing 20 µL of lysis buffer per cell. The lysis buffer contained 0.25 µL RNasin® inhibitor (Promega, Madison, WI, USA), 5 µL 5 × first-strand buffer, 1.25 µL 0.1 M DTT, and 0.0625 µL IGEPAL (Sigma-Aldrich, St. Louis, MO, USA). PCR plates containing the sorted cells were frozen on dry ice then stored at – 80°C or used for reverse transcription (RT). RT–PCR was performed and the Ig genes were cloned into expression vectors as described previously [19]. Briefly, RT was carried out by adding 1 µL of random hexamers (150 ng/µL; Promega), 2 µL dNTPs (each at 10 mM), and 0.5 µL SuperScript III (Invitrogen) to each well, followed by incubation at 42°C for 1 h. IgG heavy and light chain variable regions were independently amplified with nested PCR. The first round of PCR was performed with 2 µL of cDNA directly following RT with HotStarTaq *Plus* DNA polymerase (Qiagen, Hilden, Germany) and the primer mixtures. PCR was performed as follows: 1 cycle at 94°C for 5 min, 50 cycles at 94°C for 30 s, 55°C (round 1) or 60°C (round 2) for 30 s, and 72°C for 1 min, followed by 72°C for 10 min before cooling to 4°C. The PCR products were evaluated on 2% agarose gels. Regions displaying bands of the expected size (nearly 500 and 450 bp for the heavy chain and kappa/lambda chains, respectively) were excised from the gels and sent for Sanger sequencing after purification. Human antibody sequences were analysed using IMGT/V-QUEST (http://www.imgt.org/). Full-length IgG1 was expressed by co-transfecting HEK-293T cells with equal quantities of paired heavy and light chain plasmids based on the backbone of the pCI-neo vector [25]. Culture media were harvested four days after transfection and purified using protein A agarose (GE Healthcare).

### Enzyme-linked immunosorbent assay (ELISA)

Nunc MaxiSorp plates (Thermo Fisher Scientific, Waltham, MA, USA) were coated with 1 µg/mL ZIKV E or EDIII protein and incubated overnight at 4°C. After blocking for 2 h, the plates were incubated with cultured media containing antibodies at a 1:2 dilution or with a serial dilution of purified mAbs with a blocking buffer. The plates were washed six times with phosphate-buffered saline (PBS). The secondary antibody (goat anti-human IgG, Cat. No. ab6858; Abcam, Cambridge, UK) was applied at a 1:5000 dilution in blocking solution. The plates were incubated at 25°C for 1 h then TMB substrate was added. Absorbance was measured at 450 nm on a BioTek Synergy HTX Multi-Mode Reader (Winooski, VT, USA).

### Neutralization assay

The neutralization activity of the antibodies was measured using a flow cytometry-based neutralization assay with Vero cells as described previously [9]. Briefly, 2 × 10^5^ cells were plated in a 24-well plate 24 h before conducting the analysis. The mAbs were serially diluted and incubated with MEM (Gibco/Thermo Fisher Scientific) containing 1% FBS (Gibco). The cells were infected with 300 µL of the mixture for 2 h at 37°C under 5% CO_2_ with shaking every 15 min. Then, 1 mL/well MEM medium containing 10% FBS was added and incubated for an additional 40 h. Infected cells were collected by trypsinization then fixed and permeabilized by incubating with fixation and permeabilization solution (BD Biosciences) on ice for 20 min. Next, the cells were stained with 2 µg/mL 4G2 antibody diluted with 1 × BD Perm/Wash™ buffer (Cat. No. 554723, BD Biosciences) then stained with 1:100 diluted anti-mouse IgG FITC on ice for 30 min. After washing, the percentage of E-positive cells was measured using BD FACSCanto™ II.

### Igh repertoire amplification and sequencing

PBMCs were isolated from 8-, 6-, 8-, and 8-mL whole blood samples collected 14, 64, 181, and 412 days after symptom onset. Total RNA was extracted using TRIzol™ LS reagent according to the manufacturer’s protocol Invitrogen. ARMS-PCR using multiplex primers (iRepertoire, Inc., Huntsville, AL, USA) was performed to amplify the IgH repertoire sequences as described previously [26,27]. Briefly, multiplex primers covering nearly all of the human VH genes (forward primers) and constant region primers covering all of the five antibody isotypes (reverse primers) were designed. The forward primers Fi (forward-in) and reverse primers Ri (reverse-in) also included Illumina paired-end sequencing communal primers B and A, respectively (Illumina, San Diego, CA, USA). After PCR amplification, the first-round PCR products were used as the template for the second round PCR reaction with Illumina sequencing using communal primers B and A. Unique barcodes introduced in the first round by the constant region primers were used to distinguish the samples. After gel purification using a QIAquick® gel extraction kit (Cat No. 28704; Qiagen), the resulting product was pooled for high-throughput 250-cycle paired-end sequencing on an Illumina HiSeq 2500 sequencer (Novogene, China).

### Viruses and recombinant proteins

ZIKV GZ02 has the same genomic sequence as the ZIKV strain currently circulating in South America and was the ZIKV strain isolated from the patient in this study [23,24]. ZIKV was isolated from the blood plasma and urine of the infected patient. The virus was passaged one time in suckling mouse brain and cultured in Vero cells to prepare the stocks, which were then stored at – 80°C until use. DENVI (Hawaii strain) was prepared in Vero cells. Recombinant ZIKV E and EDIII proteins (Cat. No. 40543-V08B4 and 40543-V08B1) were purchased from Sino Biological Inc. (Beijing, China). The E protein was derived from ZIKV strain SPH2015 (KU321639), which was isolated in Brazil in 2015. DENVI EDIII protein (Cat. No. 40531-V08B) was also purchased from Sino Biological.

### Bioinformatics analysis

The Illumina 2 × 250 bp paired-end reads were extracted according to the unique barcodes at the 5′ end of the reads corresponding to the IgH constant region. Barcode-matched reads were separated and assembled based on the overlapping regions using FLASH [28]. The sequences with 300–500 nucleotides were then submitted to IMGT/HighV-QUEST [29] (http://www.imgt.org/HighV-QUEST/) to identify the V, D, and J genes. The sequences were also analysed with an in-house implementation of IgBlast [30] (http://www.ncbi.nlm.nih.gov/igblast/) to verify the accuracy of the IMGT/HighV-QUEST results. Non-productive sequences that had either stop codons or out-of-frame IGHJ were excluded from further analysis. Ig isotype proportion and global VH gene usage were calculated using the R package alakazam [31]. To determine whether the IgH repertoires contained sequences related to the antibodies we cloned, we first identified the sequences sharing the same VH and JH gene segments. Then we applied CD-HIT [32] to find the HCDR3 sequences that were 100% identical to the HCDR3 sequences of the cloned antibodies. To track the ontology and development of the isolated antibodies longitudinally, sequence identity relative to the isolated antibodies was measured via MAFFT [33] and the values of divergence from germline VH genes were calculated according to the IMGT/HighV-QUEST results. Identity-divergence plots were generated as described by Wu et al. [19].

### Tree-map analysis and diversity index D50

The CDR3 tree-map analysis and D50 calculation were performed as described by Yang et al. [26]. To draw the CDR3 tree-map for each repertoire, a set of rectangles, each of which corresponding to a distinct V gene segment in the entire repertoire was drawn, followed by a set of V-J rectangles within each V gene rectangle. Finally, within each V-J rectangle, V-J-CDR3 rectangles representing distinct V-J-CDR3 combinations were created. The V-J-CDR3 rectangles were ordered from the largest at the bottom right side to the smallest at the top left side. Therefore, each rectangle represents a cluster of nucleotide sequences with the same V gene and J gene, and identical CDR3. The size of an individual V-J-CDR3 rectangle is proportional to the relative frequency for each V-J-CDR3 combination sequence. Different rectangles were displayed in different randomly generated colours. The top 10,000 most frequent CDR3 sequences in the IgH transcripts were used for the tree-maps. Diversity index D50 is a measurement of the diversity of an immune repertoire. *J* represents the total number of CDR3s and *S* represents unique CDR3s in a ranked dominance configuration. The abundance of the most abundant CDR3 is represented by *r*_1_, while *r*_2_ is the abundance of the second most abundant CDR3 and so forth. *C* is the minimum number of distinct CDR3s with ≥ 50% total sequencing reads. Accordingly, D50 is calculated by the following equation:

$$\begin{array}{rl} & \mathrm{Assume}\;\mathrm{that}\;\underset{S}{\underbrace{r1\geq r2\cdots \geq \mathrm{ri}\cdots \geq \mathrm{ri}+1\cdots \geq \mathrm{rs}}},\;\\ & \sum_{i=1}^{S}\mathrm{ri}=J\mathrm{if}\sum_{i=1}^{c}\mathrm{ri}\geq \frac{J}{2}\mathrm{and}\sum_{i=1}^{C-1}\mathrm{ri}<\frac{J}{2}D50=\frac{C}{S}\times 100 \end{array}$$

## Results

### E-binding mabs with neutralizing activities against ZIKV were cloned from the memory B cells of a ZIKV-infected patient

Using single-cell PCR amplification of immunoglobulin heavy and light chains, we cloned 31 E protein-binding mAbs from the memory B cells of a ZIKV-infected patient 64 days after symptom onset (Table 1, Figure S1). These memory B cells were isolated using the ZIKV E protein as the probe. Fourteen E-binding mAbs, including five out of seven EDIII-targeted mAbs (7B3, 1C11, 8D10, 6B6, and 7F4) and nine out of twenty-four EDI/II-targeted mAbs (6A6, 6A11, 1E7, 6A5, 1E6, 6A9, 6B1, 6C2, and 5D2) demonstrated neutralizing activities at a concentrations lower than 5 µg/mL against the ZIKV GZ02 strain in a cell-based neutralizing assay (Table 1). Seven mAbs were bound to EDIII, indicating that they were EDIII-targeted antibodies. Detailed characterizations of some of these E-binding mAbs have been described previously [23].

**Table 1.** Summary of E-binding antibodies

ID	VH allele	DH allele	JH allele	VH SHM rate (%)	CDR3 length	HCDR3(aa)	EDIII-binding	Neutralizing activity
7B3	1-2*02	3-16*02	4*02	4.51	21	CARATDSGYDYVWGSFRYHFDSW	Yes	Yes
1C11	3-23*04	3-16*01	4*02	3.47	15	CAKDRIVLGLELFDSW	Yes	Yes
7F4	3-33*04	2-15*01	4*02	6.45	18	CARGPGWEQLQSLAAYFDFW	Yes	Yes
8D10	4-31*03	3-10*01	3*02	3.44	22	CARGLPLNYYYGSGTTALGAFDIW	Yes	Yes
6B6	4-4*02	4-17*01	4*02	3.47	12	CARDHGDLTPFEYW	Yes	Yes
6D6	3-9*01	2-2*01	6*02	3.82	23	CAKDRGVLEAGAISNYYFYYGMDVW	Yes	No
6F1	3-48*03	4-11*01	1*01	4.17	12	CARDYSTRGRFQYW	Yes	No
6A6	5-10-1*01	2-8*01	4*02	4.17	13	CVRQEDTKGRSFEYW	No	Yes
7F8	3-49*04	2-15*01	6*03	6.12	22	CTRDYCAGSSCSLQPPFYYYMDLW	No	No
7F9	4-39*02	4-11*01	5*01	7.56	10	CARQEKNWFDSW	No	No
7G11	4-39*01	1-1*01	5*01	12.71	10	CVRQDRNWFDFW	No	No
8G10	1-46*01	3-3*01	6*03	6.6	23	CARGGTIFGVVTATNYYYYYYMDVW	No	No
9C12	3-23*04	3-22*01	1*01	8.33	18	CAITIWSADSADSVGAFQYW	No	No
9G5	3-23*04	1-26*01	1*01	10.07	18	CASTIWSADSRGPVGAFQDW	No	No
1C12	4-39*02	4-11*01	5*01	12.71	10	CVRQEKNWFDSW	No	No
1D9	4-39*01	1-1*01	5*01	10.31	10	CVRQDKNWFDSW	No	No
1E6	4-61*01	3-10*01	6*03	1.72	18	CARVAGTGNSFYYYYYMDVW	No	Yes
1E7	4-39*02	4-11*01	5*01	13.06	10	CVRQEKNWFDSW	No	Yes
1G10	3-23*04	3-22*01	3*01	4.51	15	CAKDRILVVISGAFDVW	No	No
5D2	3-30*03	2-2*01	3*02	4.86	22	CARDPQGYCGSSTTCYAVGAFDIW	No	Yes
6A3	3-66*02	2-15*01	6*02	9.47	18	CAKAYCSGGVCYGYYGLDSW	No	No
6A5	4-39*02	4-11*01	5*01	14.78	10	CVRQEKNWFDSW	No	Yes
6A9	5-10-1*01	2-8*01	4*02	4.51	11	CARLTIGNDFDYW	No	Yes
6A11	3-23*04	3-22*01	1*01	4.86	20	CAKGDTYYYDATGHYLEYFQHW	No	Yes
6B1	4-31*03	3-10*01	4*02	6.53	20	CARAYGSGSYYTHDKIYYYDFW	No	Yes
6C2	4-31*03	3-10*01	4*02	7.22	20	CARAYGSGSYYTHDKIYYYDFW	No	Yes
6C10	2-5*02	4-17*01	4*02	5.15	13	CAHRLYGDLDAFDYW	No	No
6D12	4-39*02	4-11*01	5*01	9.97	10	CVRQEKNWFDSW	No	No
6E9	1-18*01	6-13*01	4*02	5.56	12	CARGTYSSTPNDYW	No	No
6G1	1-8*01	3-10*01	5*02	9.72	19	CARAEYIYGAGTEYNGWFERW	No	No
6G3	5-10-1*01	2-2*01	4*02	4.51	14	CARQVCSSSSCNLDFW	No	No

### IgH repertoires showed dramatic dominant clonal expansion at the early stages of infection but recessed to a steady state by six months

**Figure 1.**
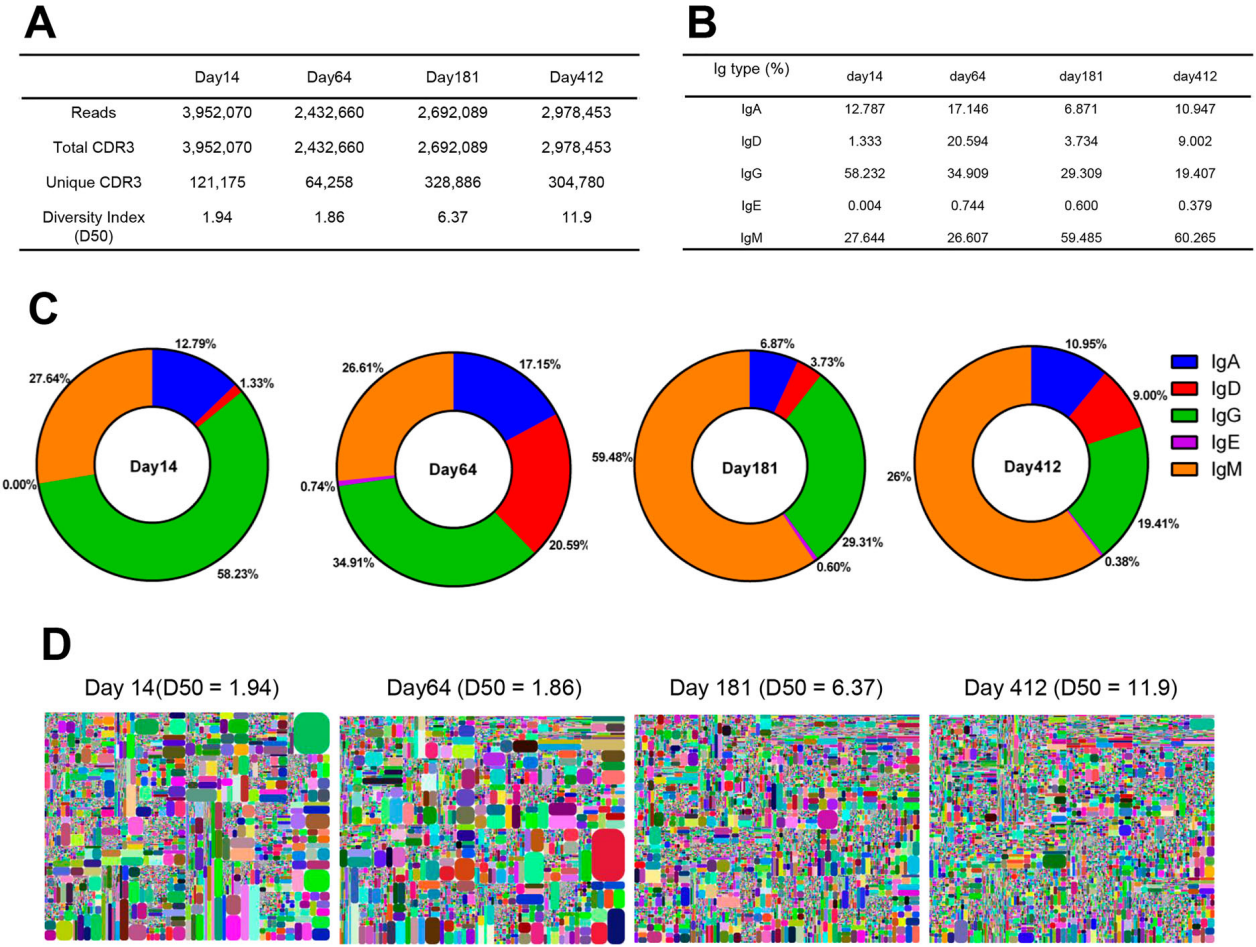
Analysis of the IgH repertoires of a ZIKV-infected patient at 14, 64, 181, and 412 days after symptom onset. (A) The total sequencing reads, total CDR3, unique CDR3, and CDR3 diversity index (D50) of the IgH repertoires. (B) The Ig subtypes were isolated and counted based on the constant sequence. Listed are the percentages of IgA, IgD, IgE, IgM, and IgG. (C) The percentages of each Ig subtypes were presented in a pie chart. (D) The V-J-CDR3 tree-map and CDR3 diversity index (D50) of the IgH repertoires. Each rectangle in the tree-map represents a unique V-J-CDR3 sequence. The size of each rectangle denotes the relative frequency of an individual V-J-CDR3 sequence. The colour of the individual CDRH3 sequence in each tree-map plot was randomly chosen; thus, the colours do not match between plots. The D50 metric value indicates the diversity of the antibody repertoire.

To understand the dynamics of the antibody response to ZIKV infection in an individual, we used iRepertoire ARMS-PCR primers for unbiased amplification of the IgH mRNA transcripts in PBMCs. Between 2.4 and 3.9 million IgH sequence reads were obtained from the samples at each timepoint by NGS using an Illumina HiSeq 2500 sequencer (Figure 1A). During sample collection, the patient was serologically confirmed to have no DENV infection [34]. The day-14 repertoire contained the highest percentage of IgG transcripts, accounting for 58% of the total, which then decreased to 35%, 29%, and 19% of the total on days 64, 181, and 412, respectively. In contrast, the IgM transcripts were lowest in the 14- and 64-day repertoires, accounting for 27.6% and 26.6% of the total, respectively; however, the IgM transcripts increased on days 181 and 412–59.5% and 60.3%, respectively. As expected, the IgE abundance was very low at all time points. Interestingly, the IgD and IgA transcripts underwent a transient increase on day 64 (Figure 1B, 1C). To obtain a holistic view of the antibody repertoire dynamics over time, we constructed four tree-maps, each representing the 10,000 most frequent unique CDR3 sequences on days 14, 64, 181, or 412 (Figure 1D). Each rectangle in a tree-map represents a unique CDR3 and its size is proportional to the relative abundance of an individual CDR3 sequence at that timepoint. On day 14, the IgH repertoire appeared to contain many dominant clones, as demonstrated by the presence of many large rectangles in the corresponding tree-map. The IgH repertoire continued to show clonal expansion until at least day 64. The tree-map of the day-64 repertoire was similar to that of the day-14 repertoire and appeared very different from the tree-maps of the day-181 and day-412 repertoires. The dominant clone content of the antibody repertoire recessed from day 64 to day 181, as demonstrated by far fewer large rectangles in the day-181 repertoire. Notably, ZIKV RNA was detected in the patient’s urine 13 days after disease onset, but it was undetectable at day 14 (Figure S1). The tree-map of the day-181 repertoire was similar to that of the day-412 repertoire, indicating that the antibody response to ZIKV ceased before day 181 (Figure 1D).

To assess the IgH repertoire diversity, we calculated the CDR3 diversity index (D50), which reflects the degree of clonal diversity [26]. The D50 values on day 14 and day 64 were the lowest (1.94 and 1.86, respectively). The D50 value increased to 6.37 on day 181 and 11.9 on day 412 (Figure 1D). Further evidence of lower antibody diversity during the early stages of infection was obtained by calculating the relative abundance of unique CDR3 sequences among the total CDR3 reads. The relative abundance of unique CDR3 sequences was lower in the day-14 (3.07%) and day-64 (2.64%) repertoires than in those of the day-181 (12.22%) and day-412 (10.23%) repertoires (Figure 1A). Taken together, these results illustrated that the antibody repertoire was largely committed to responding to the virus after ZIKV infection and lasted at least until day 64. However, when the ZIKV threat was over, the antibody repertoire recessed to a quiescent steady state with fewer dominant clones, but greater clonal diversity.

### E-binding antibodies employed diverse VH genes, while preferentially enlisting several VH genes for a rapid response to the ZIKV infection

**Figure 2.**
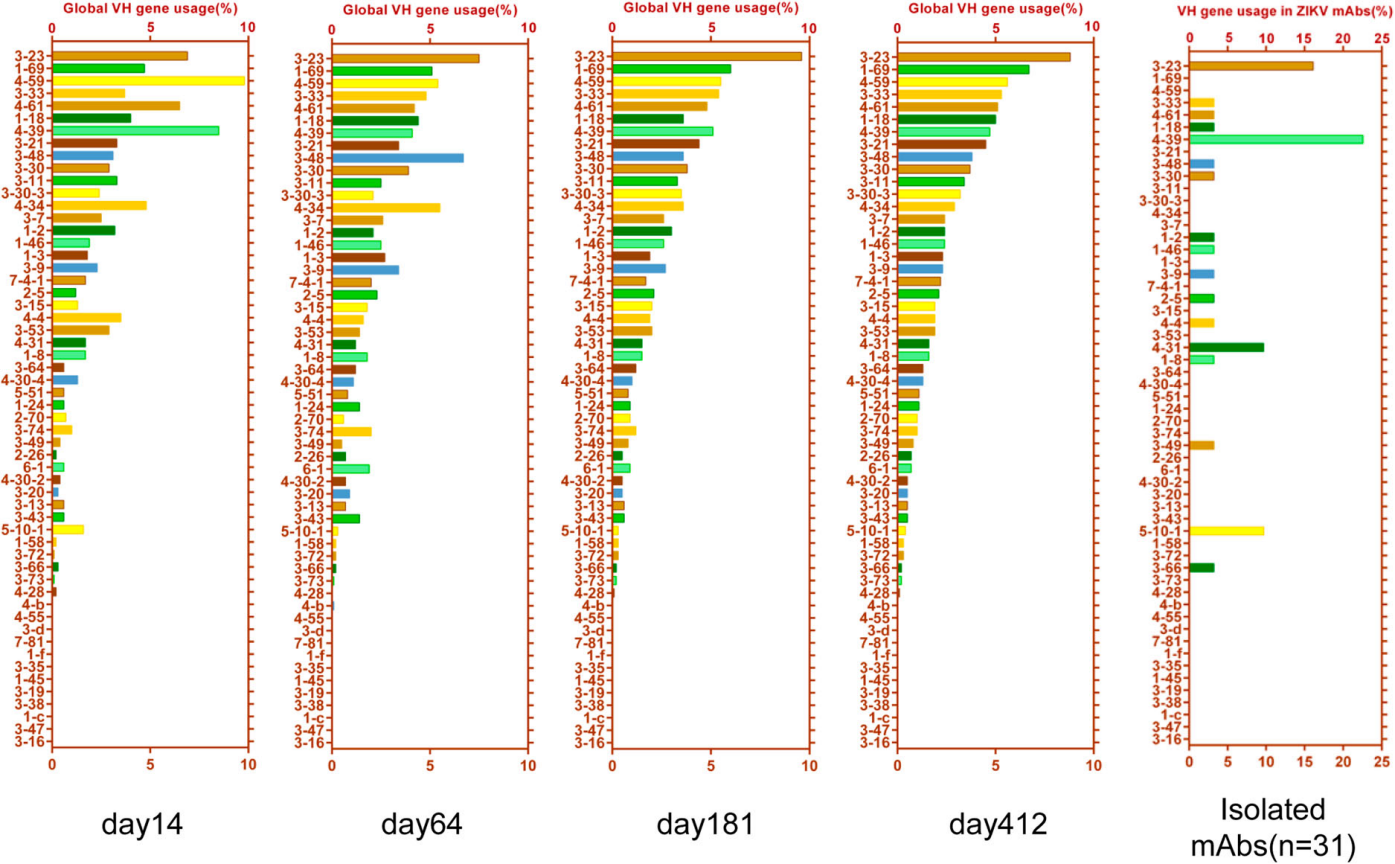
Analysis of the VH gene usage in the IgH repertoires and E-binding antibodies. The frequency of global VH gene usages in the repertoires obtained at days 14, 64, 181, and 412 were calculated. The frequency of VH gene usages is listed in descending order with respect to the frequency on day 412. The VH gene usages of 31 E-binding mAbs are shown.

We analysed the IgH sequences of the 31 E-binding mAbs that we cloned. These mAbs were derived from 17 different VH genes (Table 1). We assessed the frequency of global VH gene usage in the IgH repertoires on days 14, 64, 181, and 412 (Figure 2). The frequency of each VH gene used to generate their corresponding IgH sequences was ranked from high to low based on the sequencing data of the day-412 IgH repertoire. Since day 412 was the last timepoint collected after the patient recovered, we assumed that the corresponding repertoire reflected the most quiescent state. Indeed, the frequency of global VH gene usage in the day-181 repertoire appeared very similar to that in the day-412 repertoire, suggesting that the antibody repertoire nearly reached the quiescent state by day 181 (Figure 2). In contrast, the frequency of global VH gene usage on day 14 was very different from that on days 181 and day 412. Notably, several VH genes appeared to be more frequently used on day 14.

To determine whether any of these VH genes were preferentially enlisted to respond to the ZIKV infection, we grouped the VH gene usages for the 31 E-binding mAbs that we cloned (Figure 2, Table 1). Among the VH genes that generated E-binding mAbs, VH4-39 had the highest representation, contributing to 7 mAbs (22.58%) among the 31 mAbs that we cloned. The frequency of VH4-39 global usage was the highest in the day-14 repertoire (8.5%), coinciding with the fact that VH4-39 contributed to 7 E-binding mAbs (22.6%) that we cloned (Figure 2). However, the frequency of usage in the day-64, -181, and -412 repertoires appeared to return to basal levels of 4.1%, 4,7%, and 5.1%, respectively. VH3-23 also had a somewhat higher frequency of usage, contributing to 5 E-binding mAbs (16.1%). Most interestingly, although VH5-10-1 had an extremely low frequency of global usage (0.32%) in the day-64, -181, and -412 repertoires, its usage transiently increased five-fold to 1.6% in the day-14 repertoire (Figure 2), indicating its transient activation in response to ZIKV infection. Indeed, VH5-10-1 also had a higher representation (9.7%) among the E-binding mAbs that we cloned (Figure 2), given its extremely low frequency of global usage (0.32%) in the non-infection state reservoirs. In contrast, some VH genes such as VH1-69 which is frequently used for antibodies in response to influenza virus infection, did not contribute to any of the E-binding mAbs that we cloned, despite the fact that VH1-69 had a relatively high frequency of global usage in the day-14 repertoire (4.7%) and day-412 repertoire (6.7%).

### Most E-binding mabs had no higher SHM rates than the average SHM rates of the same VH gene-derived IgH sequences

**Figure 3.**
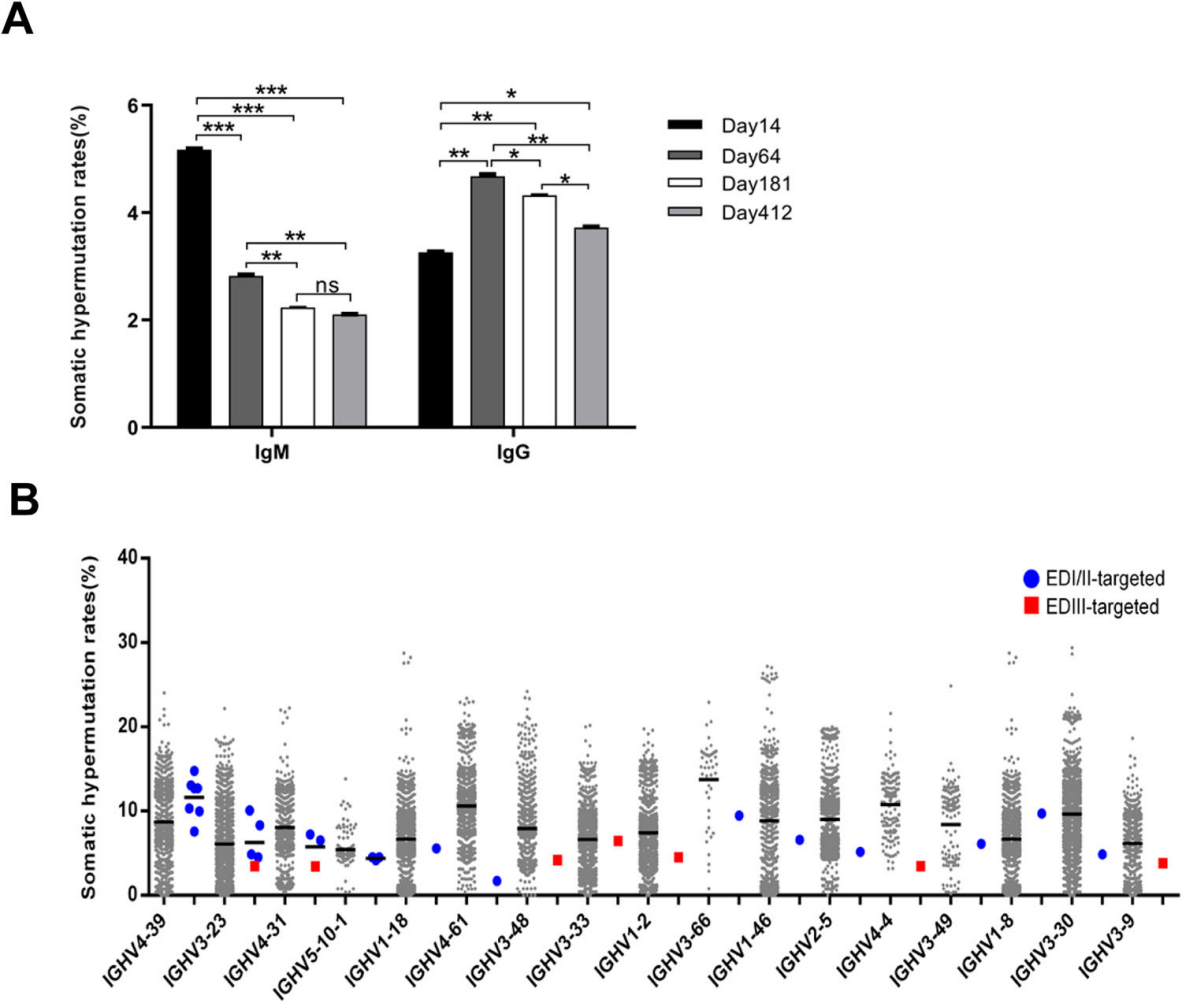
Somatic hypermutation (SHM) rates in IgH repertoires and E-binding antibodies. (A) The average VH SHM rates of IgH sequences for IgM and IgG in the day-14, -64, -181, and -412 repertoires. The SHM rates are presented as mean ± SEM. Comparison between different groups was performed by one-way ANOVA. ***p < 0.001, **p < 0.01, *p < 0.05. (B) Plot showing the level of VH SHM rates of the E-binding mAbs and all of the IgH sequences derived from the same VH gene in the day-64 repertoire. All of the 31 E-binding mAbs are presented with a solid blue circle (EDI/II-targeted mAbs) and solid red rectangle (EDIII-targeted mAbs).

We analysed the global SHM rates of IgM and IgG in the repertoires. The global SHM rates of all IgM sequences were the highest at 5.17% in the day-14 repertoire, but decreased to 2.82% on day 64 and thereafter. The global SHM rates of all IgG sequences were 3.25% on day 14, which then increased to 4.68% on day 64 then slightly decreased to 3.72% on day 412 (Figure 3A). We also analysed the SHM rates of 31 E-binding mAbs and compared these rates with those of their corresponding VH gene-derived IgH sequences in the repertoires (Table 1). The average SHM rate of the 31 E-binding mAbs was 6.91% (1.72 − 14.78%), with changes in 10.9 amino acids on average. Interestingly, the average SHM rate of 7 EDIII-targeted mAbs (7B3, 1C11, 7F4, 8D10, 6B6, 6D6, and 6F1) was 4.19% (3.44 − 6.45%), with changes in 7.5 amino acids on average. We next compared the SHM rates of each mAb to all IgH sequences derived from the same VH gene in the repertoires (Figure 3B). The SHM rates of 23 out of 31 E-binding mAbs, including all 7 EDIII-targeted mAbs, were not higher than the average SHM rates of all IgH sequences derived from the same VH gene (Figure 3B). Notably, the average SHM rate of 3 VH5-10-1-derived mAbs (6A6, 6A9, and 6G3) was 4.40% (4.17 − 4.51%). The average SHM rate of 3 VH4-31-derived mAbs (8D10, 6B1, and 6C2) was 5.73% (3.44 − 7.22%). These were all lower than the average SHM rate of all IgH sequences derived from VH5-10-1 (5.43%) and VH4-31 (8.05%) (Figure 3B, Table 1). This result indicated that most E-binding antibodies, including EDIII-targeted antibodies, could be generated without high SHM rates, suggesting a potential mechanism for the rapid response to ZIKV infection.

### B cells expressing E-binding antibodies were abundantly present in peripheral blood soon after ZIKV infection but disappeared quickly after the clearance of ZIKV

**Figure 4.**
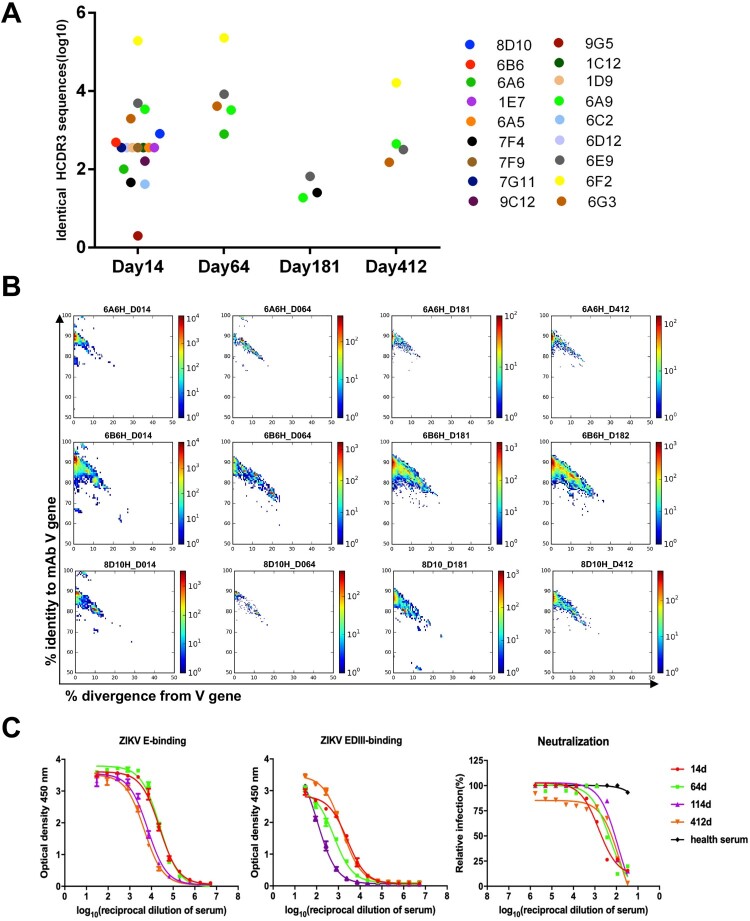
Analysis of E-binding antibodies and their evolution in the IgH repertoires. (A) The presence of sequences with the same V-J combination and CDR3 sequence as those of E-binding mAbs in the IgH repertoire on days 14, 64, 181, and 412. (B) Two-dimensional (2D) identity-divergence analysis of mAb heavy chain populations in the IgH repertoires. The heavy chain was plotted as a function of sequence similarity to the template and sequence divergence from the putative germline gene. The x-axis represents the percentage of diversity from VH genes 6A6H for VH5-10-1, 6B6H for VH4-4, and 8D10H for VH4-31 germline. The sequences are shown as a heatmap with the indicated colours. (C) The serum samples were collected on days 14, 64, 114, and 412 after disease onset. The binding activities to ZIKV E and EDIII and neutralizing activity to the ZIKV GZ02 strain were determined.

We searched the presence of IgH sequences in the reservoirs that have the same V-J gene combination and CDR3 (V-J-CDR3) sequences identical to the 31 E-binding mAbs that we cloned (Figure 4A). It was estimated that an adult person has approximately 5000 mL of blood [35]. Since the B cells that we sequenced were isolated from 6–8 mL of peripheral blood, the B cells harbouring the same V-J-CDR3 sequences may have been missed in the limited blood sample volumes that we obtained. To our surprise, 17 out of 31 E-binding mAbs with the same V-J -CDR3 sequences were found in the day-14 repertoire, whereas only 4, 3, and 3 mAbs with the same V-J-CDR3 sequences were found in the day-64, -181, and -412 repertoires, respectively (Figure 4A). This result indicated that the B cells producing E-binding antibodies were abundantly present in peripheral blood soon after infection, while these B cells quickly disappeared after the clearance of ZIKV infection. It is important to note that the 31 E-binding mAbs were cloned from the memory B cells in PBMCs collected on day 64. We next performed a two-dimensional identity-divergence analysis by searching the IgH lineage sequences of three representative mAbs (6A6, 6B6, and 8D10) in the repertoires corresponding to each time point. These three mAbs were neutralizing antibodies and their V-J-CDR3 sequences were found on day 14 but not thereafter, indicating that these mAbs were abundant on day 14 (Figure 4B). IgH lineage sequences that are highly homologous with mAb 6A6 were found in the day-14 and day-64 repertoires, but not in the day-181 and day-412 repertoires. IgH sequences that are highly homologous with mAbs 6B6 and 8D10 were found only in the day-14 repertoire (Figure 4B). Of note, mAbs 6A6, 6B6, and 8D10 all have low SHM rates of 4.17%, 3.47%, and 3.44%, respectively.

To verify whether the changes in E-binding mAbs and their closely related sequences in the IgH reservoirs correlate with the presence of E-binding antibodies in the patient’s serum, we measured the titres of E- and EDIII-binding antibodies and their neutralizing activities for serum samples collected on days 14, 64, 114, and 412 after the onset of symptoms (Figure 4C). The titre of E-binding antibodies was at the highest level on days 14 and 64 but decreased on days 114 and 412. This result is in line with the dynamic changes in the IgH repertoires revealed by the tree-maps, which showed dominant clonal expansion on days 14 and 64 (Figure 1C). Therefore, many of these clones appeared to have emerged in response to the ZIKV infection. Notably, the ZIKV neutralizing activities and EDIII-targeted antibodies were at the highest level on day 14, then declined on day 64 onward (Figure 4C). This result correlated well with the NGS analysis for the neutralizing IgH sequences in the repertoires.

Among the 17 E-binding mAbs with related V-J-CDR3 sequences found in the day-14 repertoire, 8 of them are neutralizing mAbs (8D10, 6B6, 7F4, 6A6, 1E7, 6A5, 6A9, and 6C2) and 4 of them are EDIII-targeted mAbs (8D10, 6B6, 6A6, and 7F4) (Figure 4A). However, the V-J-CDR3 sequences of only 2 neutralizing mAbs and 1 EDIII-targeted mAbs were found in the day-64 repertoire. Furthermore, the V-J-CDR3 sequences of only one neutralizing mAb and no EDIII-binding mAb were found in the day-181 and day-412 repertoires (Figure 4A). This observation suggested that EDIII-binding antibodies, as well as neutralizing antibodies, likely receded more quickly than the EDI/II-binding antibodies in this patient.

## Discussion

In this study, NGS of IgH mRNA transcripts in PBMCs in combination with single-cell cloning of ZIKV E-binding antibodies was used to analyse IgH repertoires in a patient. In the meantime, 31 heavy and light chain-paired E-binding mAbs were cloned from the memory B cells collected 64 days after the onset of symptoms. Notably, near half of these E-binding mAbs (45.2%) exhibited neutralizing activities against ZIKV. The NGS data was analysed in combination with the study of these E-binding mAbs and neutralizing mAbs.

We presented a holistic view of the antibody repertoires by drawing tree-maps to reveal the dynamic changes of the antibody response to ZIKV infection. During “wartime” and the aftermath (the first two months after ZIKV infection), there was a dramatic expansion of dominant B cell clones in response to ZIKV infection; therefore, clonal diversity was lower. During “peacetime” (six months and one year after disease), the antibody repertoire recessed to a state involving few dominant B cell clones and greater B cell clonal diversity. Given that heavy chain antibodies play a key role in antibody binding, the contribution of the light chain was not assessed in the present study. However, this may constitute a worthwhile investigation in the future. It is important to note that the patient was verified to be DENV-negative during sample collection and showed no cross-reactivity to DENV E protein for serum samples collected between two months and two years post-ZIKV infection [34], indicating that this individual had no DENV infection prior to or after the primary ZIKV infection. We inferred that if this individual manifested a DENV infection after recovery from ZIKV infection, the IgH repertoires would likely reveal changes such as the dramatic expansion of dominant B cell clones in response to a new infection. However, the IgH repertoires on day 181 and day 412 showed fewer dominant B cell clones with higher clonal diversity than the IgH repertoires on day 14 and day 64.

One limitation of this study is that the antibody repertoires for which the IgH mRNA transcripts were obtained originated from 6 to 8-ml blood samples, representing only a small fraction of the total blood present in the human body. Each unique sequence represents a B cell clone. B cells containing the exact or same lineage of IgH sequences may have been missed in the blood samples collected. Although impossible for a living subject, a complete antibody repertoire should ideally come from sequencing all B cells in an individual. Our study demonstrated that even 6–8 mL of peripheral blood can provide insightful information about antibody response. Among the 31 E-binding mAbs that we cloned from this single individual, 17 mAbs with the same V-J- CDR3 sequences could be found in the day-14 repertoire, whereas only 4 and 3 mAbs with the same V-J-CDR3 sequences could be found in the day-64 and day-181 repertoires, respectively. This finding demonstrated that B cells expressing these E-binding mAbs or their closely related lineage clones were present soon after ZIKV infection. A recent analysis of serum antibody epitope repertoires of ZIKV-infected patients also revealed that E-binding and EDIII-targeted antibodies were present early, even on the day of symptom onset, and matured with increasing affinity as early as day 7 after symptom onset [36]. This result supported our NGS analysis that B cells expressing E-binding and EDIII-targeted antibodies were abundantly present soon after ZIKV infection.

Analysis of neutralizing mAbs revealed that they were mostly present alongside its lineage divergent clones on days 14 and then 64, but not on days 181 and 412. This observation suggested that after primary ZIKV infection, B cells containing diverse VH genes underwent rapid clonal expansion upon stimulation by ZIKV antigens. B cells expressing E-binding antibodies appeared early and present in abundance in the antibody repertoire. The transient decrease of IgH clonal diversity indicated that the antibody response was largely modulated to defend against the intruding ZIKV. However, upon clearance of the virus, the antibody repertoire gradually recessed to a quiescent steady state, with few dominant B cell clones but greater diversity, conferring more effective preparation against other potential infections.

Another finding of our study is that the IgH sequences of most E-binding mAbs, including all EDIII-targeted mAbs, have relatively low SHM rates. The SHM rates of 74% of E-binding mAbs and 100% of 7 EDIII-targeted mAbs were not higher than the average SHM rate of their corresponding VH gene-derived IgH sequences (Figure 3B). Human antibodies with low SHM rates against ZIKV have been reported previously but lack of further investigation [9,11,12]. One group reported the cloning of a germline-like mAb using a phage display from a naïve human antibody library [37]. Another group reported that a panel of germline-like antibodies was cloned from circulating plasmablasts of a ZIKV-infected patient 12 days after symptom onset [38]. These mAbs exhibited weak-to-moderate neutralizing potency. One mAb had no SHM capacity, but could nevertheless bind to an immunodominant epitope and neutralize ZIKV [38]. Most of the E-binding mAbs that we cloned, including all of the EDIII-targeted mAbs, had low SHM rates (Figure 3B).

It would have been ideal if we could have obtained PBMCs from this patient before his infection, however, it would be impossible to predict who will be infected prior to infection. Nevertheless, we gathered that the PBMCs from day 412 could be considered the “no infection state.” Based on our NGS analysis of the IgH repertoire, we obtained the frequency of global VH gene usage in the same subject from early infection until over one year after recovery. Diverse VH genes were used to generate E-binding mAbs; i.e. 17 VH genes contributed to 31 mAbs that we cloned from memory B cells. However, several VH genes, such as VH4-39 and VH3-23, had a high frequency of global VH gene usage at all timepoints and a high frequency of representation among the E-binding mAbs. The contribution of the VH5-10-1 gene was particularly interesting. As in the general population [11], VH5-10-1 had a very low frequency of global VH gene usage in this patient. In this study, although VH5-10-1 had a very low frequency of global usage (average 0.32%) in the day-64, -181, and -412 repertoires, its usage on day 14 after ZIKV infection transiently elevated five-fold to 1.6%. Since VH5-10-1 had a high percentage of representation among the E-binding mAbs and low SHM rates (Table 1), we propose that VH5-10-1 was likely preferentially enlisted for rapid response to ZIKV infection in this patient. Such rapid response required only low level SHM activity; therefore, these antibodies do not require a long maturation process to achieve affinity. In this study, all three VH5-10-1-derived mAbs (6A6, 6A9, and 6G3) had low SHM rates ranging between 4.17% and 4.51% (Figure 3B, Table 1). ZIKV specific mAbs derived from VH5-10-1 has also been reported in other patients in response to ZIKV infection. For instance, another group reported that VH5-10-1 was frequently used by ZIKV-specific antibodies in DENV-ZIKV cross-infected patients and in primary ZIKV-infected patients [11]. In comparison, VH1-69 had a high frequency of global usage, but it did not contribute to any E-binding mAbs that we cloned. Among ZIKV E-binding mAbs reported by other researchers, VH1-69 was also rarely used. Since VH1-69 is frequently used in antibodies against the influenza virus [39,40], it is likely that VH1-69 is preferentially enlisted for responding to pathogens like the influenza virus but not ZIKV. In our study, by combining single B cell antibody cloning and longitudinal analysis of IgH repertoires in the same patient, we found that several VH genes such as VH5-10-1, VH4-39, and VH3-23 appeared to be preferentially enlisted for the rapid generation of E-binding antibodies after ZIKV infection.

mAbs with low SHM rates have also been isolated from patients infected with Ebola virus, RSV, and MERS-CoV [41–43]. Antibodies with a large number of SHMs require frequent antigen encounters and helper T cells in the germinal centre. We proposed that as a mechanism for rapid defence against ZIKV infection, which generally manifests a short disease course, certain VH genes are preferentially selected to generate ZIKV-specific antibodies without the need to undergo a long maturation process. When a person is exposed to ZIKV infection, the primary B cell response from naïve B cells can rapidly generate ZIKV-specific antibodies, including neutralizing antibodies that do not require the accumulation of a large number of SHMs. The preferential usage of these VH genes may be attributed to evolution through many years of interaction between *Homo sapiens* and ZIKV, along with other flaviviruses. Our longitudinal analysis of IgH repertoires corroborated well with the results from single B cell antibody cloning and with the neutralizing, E-binding, and EDIII-binding activities in the serum samples collected from days 14–412. Our study showed that EDIII-targeted antibodies in the IgH reservoirs correlated well with serum-neutralizing activities against ZIKV. This result was also in line with a report that the presence of EDIII-targeted antibodies correlated with serum-neutralizing activity against ZIKV [8]. Our study also indicated that E-binding antibodies persisted longer than EDIII-binding antibodies and neutralizing antibodies in this patient, implying that EDI- and EDII-binding antibodies may last longer in the patient. Since most EDI- and EDII-binding antibodies have no or low-neutralizing activities, they may play a role in the antibody-mediated enhancement of infection of other flaviviruses that share similar epitopes. It would be worthwhile to study a large cohort of samples in the future. This study provides insight on the antibody response after ZIKV infection and has implications for the design and development of vaccines against ZIKV.

## Supplementary Material

Supplemental Material
