# Supplementary figures and images for: Longitudinal analysis of the antibody repertoire of a Zika virus-infected patient revealed dynamic changes in antibody response
Source: Emerg Microbes Infect. 2020 Jan 6;9(1):111–23. doi: 10.1080/22221751.2019.1701953 (PMC6968589; doi:10.1080/22221751.2019.1701953)

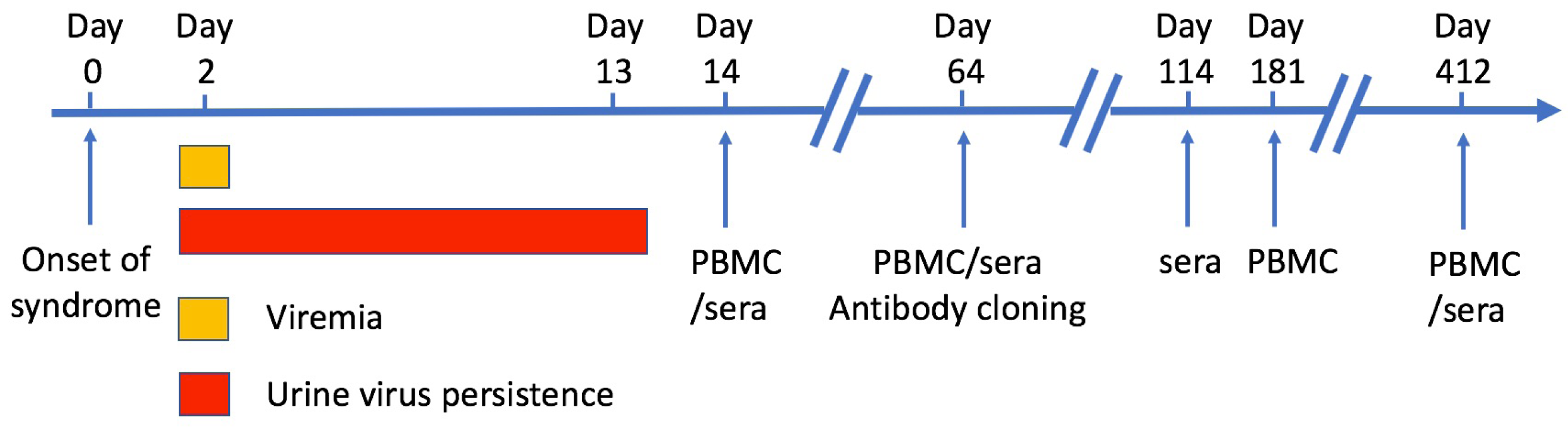

Supplement: Supplemental Material [file TEMI_A_1701953_SM4445.tif]
